# 4-(7-Acet­oxy-6-meth­oxy-4-methyl-2-oxo-2*H*-chromen-3-yl)phenyl acetate

**DOI:** 10.1107/S1600536808012890

**Published:** 2008-06-07

**Authors:** Hao Jiang, Peng Xia, Qian Zhang

**Affiliations:** a Department of Medicinal Chemistry, School of Pharmacy, Fudan University, Shanghai 200032, People’s Republic of China

## Abstract

The title compound, C_21_H_18_O_7_, is an important inter­mediate in the synthesis of 3-(4-hydroxy­phen­yl)-4-methyl-6-meth­oxy-7-hydroxy­coumarin, which is a nonsteroidal analogue of 2-methoxy­estradiol (2-ME). The substituent benzene ring is not in the same plane as the coumarin ring system, with a dihedral angle of 66.88 (10)°. There are some weak inter­molecular C—H⋯O inter­actions. One carbonyl O atom is disordered over two sites, with occupancies of 0.6 and 0.4.

## Related literature

For related literature, see: Gibanananda *et al.* (2006 ▶); Sutherland *et al.* (2007 ▶).
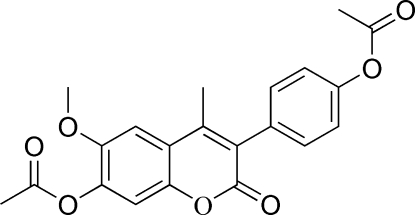


## Experimental

### 

#### Crystal data

C_21_H_18_O_7_
*M* *_r_* = 382.35
Triclinic, 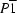

*a* = 8.142 (3) Å
*b* = 11.167 (4) Å
*c* = 11.756 (4) Å
α = 65.130 (4)°
β = 75.392 (4)°
γ = 79.055 (4)°
*V* = 934.1 (5) Å^3^
*Z* = 2
Mo *K*α radiation
μ = 0.10 mm^−1^
*T* = 293 (2) K
0.15 × 0.12 × 0.04 mm

#### Data collection

Bruker SMART APEX CCD area-detector diffractometer
Absorption correction: multi-scan (*SADABS*; Sheldrick, 1996 ▶) *T*_min_ = 0.985, *T*_max_ = 0.996
3893 measured reflections
3245 independent reflections
2279 reflections with *I* > 2σ(*I*)
*R*_int_ = 0.031

#### Refinement

*R*[*F* ^2^ > 2σ(*F* ^2^)] = 0.074
*wR*(*F* ^2^) = 0.254
*S* = 1.09
3245 reflections
266 parameters
1 restraint
H-atom parameters constrained
Δρ_max_ = 0.28 e Å^−3^
Δρ_min_ = −0.50 e Å^−3^

### 

Data collection: *SMART* (Bruker, 2000 ▶); cell refinement: *SMART*; data reduction: *SAINT* (Bruker, 2000 ▶); program(s) used to solve structure: *SHELXS97* (Sheldrick, 2008 ▶); program(s) used to refine structure: *SHELXL97* (Sheldrick, 2008 ▶); molecular graphics: *SHELXTL* (Sheldrick, 2008 ▶); software used to prepare material for publication: *SHELXTL*.

## Supplementary Material

Crystal structure: contains datablocks I, global. DOI: 10.1107/S1600536808012890/cf2199sup1.cif

Structure factors: contains datablocks I. DOI: 10.1107/S1600536808012890/cf2199Isup2.hkl

Additional supplementary materials:  crystallographic information; 3D view; checkCIF report

## Figures and Tables

**Table 1:** Hydrogen-bond geometry (Å, °)

*D*—H⋯*A*	*D*—H	H⋯*A*	*D*⋯*A*	*D*—H⋯*A*
C20—H20*B*⋯O2^i^	0.96	2.47	3.362 (4)	154
C20—H20*C*⋯O4*B*^i^	0.96	2.55	3.297 (9)	134
C11—H11*B*⋯O7^ii^	0.96	2.74	3.349 (4)	122
C13—H13⋯O2^iii^	0.93	2.74	3.331 (4)	122
C19—H19*A*⋯O7^iii^	0.96	2.50	3.392 (5)	154
C17—H17⋯O2^iv^	0.93	2.66	3.246 (3)	122

Symmetry codes: (i) 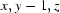; (ii) 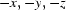; (iii) 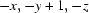; (iv) 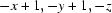.

**Fig. 1.**
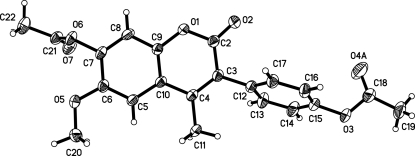
The molecular structure of (I), with atom labels and 30% probability displacement ellipsoids for non-H atoms. The minor disorder component is not shown.

**Fig. 2.**
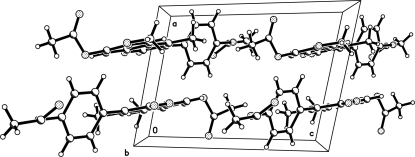
Packing diagram, viewed down the b axis.

## supplementary crystallographic information

### Comment

2-ME, an endogenous metabolite of estrogen, was proved to be a potent antitumor and antiangiogenic compound (Gibanananda *et al.*, 2006). Currently 2-ME is in phase I–III clinical trials for treating a variety of solid cancers, especially breast cancer, prostate cancer and multiple myeloma (Sutherland *et al.*, 2007). Based on the structure and the bioactivity of 2-ME, 3-(*p*-hydroxyphenyl)-4-methyl-6-methoxyl-7-hydroxycoumarin, an non-steroidal analog of 2-ME, was designed, synthesized and evaluated on Human Umbilical Vein Endothelial Cells (HUVEC). The compound showed higher activity and much lower toxicity (EC_50_ = 5.69 µ*M*; TI = 45.01) than 2-ME (EC_50_ = 8.59 µ*M*; TI = 8.25) in the biological assay. Here we report the crystal structure of 3-(*p*-acetoxyphenyl)-4-methyl-6-methoxyl-7-acetoxycoumarin, which is an important intermediate in the synthesis of 3-(*p*-hydroxyphenyl)-4-methyl-6-methoxyl-7-hydroxycoumarin. The molecular structure of (I) is illustrated in Fig.1. The coumarin ring system (C1—C10) is essentially planar, with a mean deviation of 0.0153 Å from the least-squares plane defined by the ten constituent atoms. The coumarin ring system and the 3-aryl ring make a dihedral angle of 66.88 (10)°. The fact that the of C3—C12 bond [length 1.480 (4) Å] is a single bond also confirms that the coumarin ring system and the 3-substituent are not conjugated. The molecular packing (Fig. 2) is stabilized by weak intermolecular C—H···O hydrogen bonds.

### Experimental

A mixture of 1-(2,4-dihydroxyl-5-methoxyphenyl)ethanone (300 mg, 1.65 mmol), 4-hydroxyphenylacetic acid (501 mg, 3.29 mmol), Et_3_N (6 ml) and Ac_2_O (10 ml) was refluxed for 10 h. After cooling, the mixture was poured into 2 *N* HCl (20 ml) and extracted with acetyl acetate. The organic layer was dried over Na_2_SO_4_, filtered, and concentrated under reduced pressure to give a yellow oil, which was purified *via* chromatography on silica gel column with petroleum ether/acetone (10:3) as eluent. The title compound was recrystallized from acetyl acetate to give colorless crystals for the single-crystal X-ray diffraction analysis.

### Refinement

All H atoms were positioned geometrically and refined using a riding model with C—H = 0.93 Å for aromatic H atoms and 0.96 Å for methyl H atoms, and refined in riding mode with *U*_iso_(H) = 1.2 *U*_eq_(C) for aromatic H atoms and *U*_iso_(H) = 1.5 *U*_eq_(C) for methyl H atoms.

### Crystal data

**Table:**

C_21_H_18_O_7_	*Z* = 2
*M**_r_* = 382.35	*F*_000_ = 400
Triclinic, *P*1	*D*_x_ = 1.359 Mg m^−^^3^
Hall symbol: -P 1	Mo *K*α radiation λ = 0.71073 Å
*a* = 8.142 (3) Å	Cell parameters from 954 reflections
*b* = 11.167 (4) Å	θ = 2.6–26.3º
*c* = 11.756 (4) Å	µ = 0.10 mm^−^^1^
α = 65.130 (4)º	*T* = 293 (2) K
β = 75.392 (4)º	Sheet, colorless
γ = 79.055 (4)º	0.15 × 0.12 × 0.04 mm
*V* = 934.1 (5) Å^3^	

### Data collection

**Table:**

Bruker SMART APEX CCD area-detector diffractometer	3245 independent reflections
Radiation source: fine-focus sealed tube	2279 reflections with *I* > 2σ(*I*)
Monochromator: graphite	*R*_int_ = 0.031
*T* = 293(2) K	θ_max_ = 25.1º
φ and ω scans	θ_min_ = 2.0º
Absorption correction: multi-scan(SADABS; Sheldrick, 1996)	*h* = −9→9
*T*_min_ = 0.985, *T*_max_ = 0.996	*k* = −13→10
3893 measured reflections	*l* = −13→14

### Refinement

**Table:**

Refinement on *F*^2^	Secondary atom site location: difference Fourier map
Least-squares matrix: full	Hydrogen site location: inferred from neighbouring sites
*R*[*F*^2^ > 2σ(*F*^2^)] = 0.074	H-atom parameters constrained
*wR*(*F*^2^) = 0.254	*w* = 1/[σ^2^(*F*_o_^2^) + (0.1741*P*)^2^] where *P* = (*F*_o_^2^ + 2*F*_c_^2^)/3
*S* = 1.10	(Δ/σ)_max_ < 0.001
3245 reflections	Δρ_max_ = 0.28 e Å^−^^3^
266 parameters	Δρ_min_ = −0.50 e Å^−^^3^
1 restraint	Extinction correction: none
Primary atom site location: structure-invariant direct methods	

### Special details

**Table:**

Geometry. All e.s.d.'s (except the e.s.d. in the dihedral angle between two l.s. planes) are estimated using the full covariance matrix. The cell e.s.d.'s are taken into account individually in the estimation of e.s.d.'s in distances, angles and torsion angles; correlations between e.s.d.'s in cell parameters are only used when they are defined by crystal symmetry. An approximate (isotropic) treatment of cell e.s.d.'s is used for estimating e.s.d.'s involving l.s. planes.
Refinement. Refinement of *F*^2^ against ALL reflections. The weighted *R*-factor *wR* and goodness of fit *S* are based on *F*^2^, conventional *R*-factors *R* are based on *F*, with *F* set to zero for negative *F*^2^. The threshold expression of *F*^2^ > σ(*F*^2^) is used only for calculating *R*-factors(gt) *etc*. and is not relevant to the choice of reflections for refinement. *R*-factors based on *F*^2^ are statistically about twice as large as those based on *F*, and *R*- factors based on ALL data will be even larger.

### Fractional atomic coordinates and isotropic or equivalent isotropic displacement parameters (Å^2^)

**Table:**

	*x*	*y*	*z*	*U*_iso_*/*U*_eq_	Occ. (<1)
O1	0.3185 (3)	0.17959 (18)	0.16190 (17)	0.0502 (6)	
O2	0.3094 (3)	0.3939 (2)	0.1010 (2)	0.0586 (6)	
O3	0.2256 (3)	0.8533 (2)	−0.4110 (2)	0.0687 (7)	
O5	0.2670 (3)	−0.27608 (18)	0.10771 (19)	0.0563 (6)	
O6	0.3460 (2)	−0.28758 (18)	0.31855 (17)	0.0507 (6)	
O7	0.0661 (3)	−0.3060 (2)	0.3850 (2)	0.0736 (7)	
C2	0.2997 (4)	0.3052 (3)	0.0710 (3)	0.0461 (7)	
C3	0.2680 (3)	0.3215 (3)	−0.0519 (3)	0.0422 (7)	
C4	0.2529 (3)	0.2128 (3)	−0.0733 (2)	0.0405 (6)	
C5	0.2552 (3)	−0.0362 (3)	0.0142 (2)	0.0425 (7)	
H5	0.2329	−0.0317	−0.0613	0.051*	
C6	0.2753 (3)	−0.1569 (3)	0.1113 (3)	0.0442 (7)	
C7	0.3133 (3)	−0.1642 (3)	0.2232 (3)	0.0440 (7)	
C8	0.3266 (4)	−0.0521 (3)	0.2393 (3)	0.0481 (7)	
H8	0.3507	−0.0578	0.3148	0.058*	
C9	0.3036 (3)	0.0699 (3)	0.1407 (2)	0.0413 (6)	
C10	0.2678 (3)	0.0826 (3)	0.0268 (2)	0.0399 (6)	
C11	0.2208 (4)	0.2260 (3)	−0.1983 (3)	0.0527 (8)	
H11A	0.2364	0.3147	−0.2601	0.079*	
H11B	0.1061	0.2074	−0.1871	0.079*	
H11C	0.2992	0.1644	−0.2276	0.079*	
C12	0.2578 (3)	0.4597 (3)	−0.1475 (3)	0.0444 (7)	
C13	0.1053 (4)	0.5256 (3)	−0.1852 (3)	0.0592 (8)	
H13	0.0075	0.4810	−0.1518	0.071*	
C14	0.0962 (4)	0.6549 (3)	−0.2704 (3)	0.0641 (9)	
H14	−0.0067	0.6966	−0.2953	0.077*	
C15	0.2384 (4)	0.7232 (3)	−0.3192 (3)	0.0524 (8)	
C16	0.3906 (4)	0.6615 (3)	−0.2841 (3)	0.0540 (8)	
H16	0.4875	0.7072	−0.3181	0.065*	
C17	0.3995 (4)	0.5314 (3)	−0.1982 (3)	0.0497 (7)	
H17	0.5028	0.4907	−0.1737	0.060*	
C18	0.2305 (6)	0.9555 (4)	−0.3829 (4)	0.0864 (13)	
O4A	0.1775 (16)	0.9331 (7)	−0.2672 (6)	0.159 (4)	0.55
O4B	0.3254 (11)	0.9405 (7)	−0.3076 (8)	0.094 (2)	0.45
C19	0.2078 (6)	1.0852 (3)	−0.4860 (4)	0.0876 (12)	
H19A	0.1643	1.1509	−0.4506	0.131*	
H19B	0.1287	1.0822	−0.5329	0.131*	
H19C	0.3155	1.1075	−0.5424	0.131*	
C20	0.2417 (4)	−0.2736 (3)	−0.0087 (3)	0.0571 (8)	
H20A	0.1307	−0.2306	−0.0234	0.086*	
H20B	0.2502	−0.3627	−0.0032	0.086*	
H20C	0.3270	−0.2258	−0.0781	0.086*	
C21	0.2086 (4)	−0.3556 (3)	0.3919 (3)	0.0515 (8)	
C22	0.2624 (5)	−0.4899 (3)	0.4777 (3)	0.0720 (10)	
H22A	0.2921	−0.4860	0.5500	0.108*	
H22B	0.3596	−0.5265	0.4327	0.108*	
H22C	0.1707	−0.5449	0.5065	0.108*	

### Atomic displacement parameters (Å^2^)

**Table:**

	*U*^11^	*U*^22^	*U*^33^	*U*^12^	*U*^13^	*U*^23^
O1	0.0708 (14)	0.0396 (11)	0.0404 (11)	−0.0112 (9)	−0.0212 (9)	−0.0075 (9)
O2	0.0779 (15)	0.0445 (12)	0.0597 (13)	−0.0139 (10)	−0.0268 (11)	−0.0153 (10)
O3	0.113 (2)	0.0374 (12)	0.0452 (12)	−0.0105 (12)	−0.0248 (12)	0.0010 (9)
O5	0.0827 (15)	0.0348 (11)	0.0463 (12)	−0.0121 (10)	−0.0177 (10)	−0.0054 (9)
O6	0.0515 (12)	0.0402 (11)	0.0424 (11)	−0.0096 (9)	−0.0125 (9)	0.0050 (9)
O7	0.0529 (14)	0.0680 (16)	0.0697 (16)	−0.0088 (12)	−0.0070 (11)	0.0002 (12)
C2	0.0489 (16)	0.0437 (16)	0.0429 (16)	−0.0153 (12)	−0.0139 (12)	−0.0068 (13)
C3	0.0420 (15)	0.0379 (15)	0.0410 (15)	−0.0089 (11)	−0.0109 (12)	−0.0064 (12)
C4	0.0398 (14)	0.0402 (15)	0.0345 (14)	−0.0074 (11)	−0.0094 (11)	−0.0052 (11)
C5	0.0492 (16)	0.0401 (15)	0.0345 (14)	−0.0087 (12)	−0.0120 (12)	−0.0072 (12)
C6	0.0464 (15)	0.0364 (15)	0.0426 (15)	−0.0107 (12)	−0.0077 (12)	−0.0062 (12)
C7	0.0437 (15)	0.0378 (14)	0.0361 (14)	−0.0081 (11)	−0.0069 (11)	0.0005 (11)
C8	0.0555 (17)	0.0502 (17)	0.0336 (14)	−0.0113 (14)	−0.0154 (12)	−0.0053 (12)
C9	0.0469 (15)	0.0372 (14)	0.0371 (14)	−0.0089 (11)	−0.0092 (11)	−0.0093 (11)
C10	0.0402 (14)	0.0402 (15)	0.0336 (14)	−0.0096 (11)	−0.0089 (11)	−0.0057 (11)
C11	0.073 (2)	0.0404 (15)	0.0380 (15)	−0.0088 (14)	−0.0198 (14)	−0.0027 (12)
C12	0.0499 (16)	0.0397 (15)	0.0401 (15)	−0.0092 (12)	−0.0122 (12)	−0.0084 (12)
C13	0.0510 (17)	0.0442 (17)	0.065 (2)	−0.0106 (14)	−0.0162 (15)	0.0009 (14)
C14	0.0596 (19)	0.0531 (19)	0.063 (2)	−0.0022 (15)	−0.0246 (16)	−0.0007 (15)
C15	0.072 (2)	0.0405 (16)	0.0363 (15)	−0.0119 (14)	−0.0124 (14)	−0.0031 (12)
C16	0.0615 (19)	0.0416 (16)	0.0510 (17)	−0.0156 (14)	−0.0083 (14)	−0.0075 (13)
C17	0.0490 (16)	0.0442 (16)	0.0510 (17)	−0.0111 (13)	−0.0130 (13)	−0.0092 (13)
C18	0.153 (4)	0.045 (2)	0.057 (2)	−0.003 (2)	−0.040 (3)	−0.0074 (16)
O4A	0.341 (14)	0.060 (4)	0.057 (4)	0.018 (7)	−0.045 (6)	−0.017 (3)
O4B	0.162 (7)	0.044 (3)	0.087 (5)	−0.015 (4)	−0.069 (5)	−0.009 (3)
C19	0.128 (4)	0.0412 (19)	0.070 (2)	−0.002 (2)	−0.020 (2)	−0.0011 (16)
C20	0.071 (2)	0.0475 (17)	0.0543 (18)	−0.0161 (14)	−0.0085 (15)	−0.0191 (14)
C21	0.0548 (19)	0.0492 (17)	0.0397 (16)	−0.0136 (14)	−0.0073 (13)	−0.0047 (13)
C22	0.077 (2)	0.0501 (19)	0.061 (2)	−0.0128 (17)	−0.0088 (17)	0.0060 (16)

### Geometric parameters (Å, °)

**Table:**

O1—C2	1.369 (3)	C11—H11C	0.960
O1—C9	1.380 (3)	C12—C17	1.389 (4)
O2—C2	1.205 (3)	C12—C13	1.392 (4)
O3—C18	1.324 (4)	C13—C14	1.369 (4)
O3—C15	1.402 (3)	C13—H13	0.930
O5—C6	1.364 (3)	C14—C15	1.372 (5)
O5—C20	1.423 (4)	C14—H14	0.930
O6—C21	1.363 (3)	C15—C16	1.369 (5)
O6—C7	1.392 (3)	C16—C17	1.378 (4)
O7—C21	1.194 (4)	C16—H16	0.930
C2—C3	1.461 (4)	C17—H17	0.930
C3—C4	1.371 (4)	C18—O4A	1.248 (7)
C3—C12	1.480 (4)	C18—O4B	1.257 (7)
C4—C10	1.442 (3)	C18—C19	1.461 (5)
C4—C11	1.498 (4)	C19—H19A	0.960
C5—C6	1.364 (4)	C19—H19B	0.960
C5—C10	1.421 (4)	C19—H19C	0.960
C5—H5	0.930	C20—H20A	0.960
C6—C7	1.392 (4)	C20—H20B	0.960
C7—C8	1.369 (4)	C20—H20C	0.960
C8—C9	1.386 (4)	C21—C22	1.470 (4)
C8—H8	0.930	C22—H22A	0.960
C9—C10	1.386 (4)	C22—H22B	0.960
C11—H11A	0.960	C22—H22C	0.960
C11—H11B	0.960		
			
C2—O1—C9	121.4 (2)	C14—C13—H13	119.3
C18—O3—C15	120.8 (2)	C12—C13—H13	119.3
C6—O5—C20	116.9 (2)	C13—C14—C15	120.2 (3)
C21—O6—C7	116.7 (2)	C13—C14—H14	119.9
O2—C2—O1	115.9 (2)	C15—C14—H14	119.9
O2—C2—C3	125.5 (3)	C16—C15—C14	120.0 (3)
O1—C2—C3	118.6 (2)	C16—C15—O3	121.5 (3)
C4—C3—C2	120.2 (2)	C14—C15—O3	118.4 (3)
C4—C3—C12	124.4 (2)	C15—C16—C17	119.7 (3)
C2—C3—C12	115.4 (2)	C15—C16—H16	120.1
C3—C4—C10	119.3 (2)	C17—C16—H16	120.1
C3—C4—C11	121.6 (2)	C16—C17—C12	121.6 (3)
C10—C4—C11	119.1 (2)	C16—C17—H17	119.2
C6—C5—C10	121.0 (2)	C12—C17—H17	119.2
C6—C5—H5	119.5	O4A—C18—O3	113.0 (5)
C10—C5—H5	119.5	O4B—C18—O3	117.6 (5)
O5—C6—C5	125.2 (2)	O4A—C18—C19	123.9 (5)
O5—C6—C7	115.1 (2)	O4B—C18—C19	119.1 (5)
C5—C6—C7	119.6 (2)	O3—C18—C19	114.7 (3)
C8—C7—O6	119.0 (2)	C18—C19—H19A	109.5
C8—C7—C6	121.2 (2)	C18—C19—H19B	109.5
O6—C7—C6	119.7 (2)	H19A—C19—H19B	109.5
C7—C8—C9	118.6 (2)	C18—C19—H19C	109.5
C7—C8—H8	120.7	H19A—C19—H19C	109.5
C9—C8—H8	120.7	H19B—C19—H19C	109.5
O1—C9—C10	121.3 (2)	O5—C20—H20A	109.5
O1—C9—C8	116.2 (2)	O5—C20—H20B	109.5
C10—C9—C8	122.5 (2)	H20A—C20—H20B	109.5
C9—C10—C5	117.0 (2)	O5—C20—H20C	109.5
C9—C10—C4	119.1 (2)	H20A—C20—H20C	109.5
C5—C10—C4	123.8 (2)	H20B—C20—H20C	109.5
C4—C11—H11A	109.5	O7—C21—O6	121.7 (3)
C4—C11—H11B	109.5	O7—C21—C22	127.3 (3)
H11A—C11—H11B	109.5	O6—C21—C22	111.0 (3)
C4—C11—H11C	109.5	C21—C22—H22A	109.5
H11A—C11—H11C	109.5	C21—C22—H22B	109.5
H11B—C11—H11C	109.5	H22A—C22—H22B	109.5
C17—C12—C13	117.1 (3)	C21—C22—H22C	109.5
C17—C12—C3	120.9 (2)	H22A—C22—H22C	109.5
C13—C12—C3	121.9 (2)	H22B—C22—H22C	109.5
C14—C13—C12	121.4 (3)		
			
C9—O1—C2—O2	177.5 (2)	C8—C9—C10—C4	−177.3 (2)
C9—O1—C2—C3	−1.8 (4)	C6—C5—C10—C9	0.9 (4)
O2—C2—C3—C4	−177.4 (3)	C6—C5—C10—C4	178.1 (2)
O1—C2—C3—C4	1.8 (4)	C3—C4—C10—C9	−2.5 (4)
O2—C2—C3—C12	3.8 (4)	C11—C4—C10—C9	177.7 (2)
O1—C2—C3—C12	−177.0 (2)	C3—C4—C10—C5	−179.6 (2)
C2—C3—C4—C10	0.3 (4)	C11—C4—C10—C5	0.6 (4)
C12—C3—C4—C10	179.0 (2)	C4—C3—C12—C17	−113.2 (3)
C2—C3—C4—C11	−179.9 (2)	C2—C3—C12—C17	65.6 (4)
C12—C3—C4—C11	−1.2 (4)	C4—C3—C12—C13	70.4 (4)
C20—O5—C6—C5	2.5 (4)	C2—C3—C12—C13	−110.8 (3)
C20—O5—C6—C7	−175.2 (2)	C17—C12—C13—C14	1.2 (5)
C10—C5—C6—O5	−179.4 (2)	C3—C12—C13—C14	177.8 (3)
C10—C5—C6—C7	−1.8 (4)	C12—C13—C14—C15	−1.0 (5)
C21—O6—C7—C8	106.7 (3)	C13—C14—C15—C16	0.9 (5)
C21—O6—C7—C6	−76.1 (3)	C13—C14—C15—O3	176.9 (3)
O5—C6—C7—C8	179.6 (2)	C18—O3—C15—C16	−74.4 (5)
C5—C6—C7—C8	1.7 (4)	C18—O3—C15—C14	109.6 (4)
O5—C6—C7—O6	2.5 (4)	C14—C15—C16—C17	−1.0 (5)
C5—C6—C7—O6	−175.4 (2)	O3—C15—C16—C17	−176.9 (3)
O6—C7—C8—C9	176.3 (2)	C15—C16—C17—C12	1.1 (5)
C6—C7—C8—C9	−0.8 (4)	C13—C12—C17—C16	−1.2 (4)
C2—O1—C9—C10	−0.4 (4)	C3—C12—C17—C16	−177.8 (3)
C2—O1—C9—C8	179.4 (2)	C15—O3—C18—O4A	−27.7 (9)
C7—C8—C9—O1	−180.0 (2)	C15—O3—C18—O4B	35.1 (8)
C7—C8—C9—C10	−0.1 (4)	C15—O3—C18—C19	−177.3 (3)
O1—C9—C10—C5	179.9 (2)	C7—O6—C21—O7	−8.4 (4)
C8—C9—C10—C5	0.0 (4)	C7—O6—C21—C22	173.1 (3)
O1—C9—C10—C4	2.6 (4)		

### Hydrogen-bond geometry (Å, °)

**Table:**

*D*—H···*A*	*D*—H	H···*A*	*D*···*A*	*D*—H···*A*
C20—H20B···O2^i^	0.96	2.47	3.362 (4)	154
C20—H20C···O4B^i^	0.96	2.55	3.297 (9)	134
C11—H11B···O7^ii^	0.96	2.74	3.349 (4)	122
C13—H13···O2^iii^	0.93	2.74	3.331 (4)	122
C19—H19A···O7^iii^	0.96	2.50	3.392 (5)	154
C17—H17···O2^iv^	0.93	2.66	3.246 (3)	122

Symmetry codes: (i) *x*, *y*−1, *z*; (ii) −*x*, −*y*, −*z*; (iii) −*x*, −*y*+1, −*z*; (iv) −*x*+1, −*y*+1, −*z*.
